# *In-situ* characterization of ultrathin nickel silicides using 3D medium-energy ion scattering

**DOI:** 10.1038/s41598-020-66464-1

**Published:** 2020-06-24

**Authors:** Tuan Thien Tran, Lukas Jablonka, Christian Lavoie, Zhen Zhang, Daniel Primetzhofer

**Affiliations:** 1 Department of Physics and Astronomy, Ångström Laboratory, Uppsala University, Box 516, SE-751 20, Uppsala, Sweden; 2 Solid State Electronics, The Ångström Laboratory, Uppsala University, SE-75121, Uppsala, Sweden; 3 IBM Thomas J. Watson Research Center, Yorktown Heights, New York, 10598 USA

**Keywords:** Characterization and analytical techniques, Surfaces, interfaces and thin films, Structure of solids and liquids, Phase transitions and critical phenomena

## Abstract

Epitaxial ultrathin films are of utmost importance for state-of-the-art nanoelectronic devices, such as MOSFET transistors and non-volatile memories. At the same time, as the film thickness is reduced to a few nanometers, characterization of the materials is becoming challenging for commonly used methods. In this report, we demonstrate an approach for *in-situ* characterization of phase transitions of ultrathin nickel silicides using 3D medium-energy ion scattering. The technique provides simultaneously depth-resolved composition and real-space crystallography of the silicide films using a single sample and with a non-invasive probe. We show, for 10 nm Ni films on Si, that their composition follows a normal transition sequence, such as Ni-Ni_2_Si-NiSi. However, the transition process is significantly different for samples with initial Ni thickness of 3 nm. Depth-resolved crystallography shows that the Ni films transform from an as-deposited disordered layer to an epitaxial silicide layer at the temperature of ~290 °C, significantly lower than previously reported. The high depth resolution of the technique permits us to determine the composition of the ultrathin films to be 38% Ni and 62% Si.

## Introduction

Metal silicides have been decisive compounds for the development of high-performance electronic devices. These materials enable low contact resistance between the devices and the interconnects, hence providing manifold benefits, such as higher drive current, lower power consumption and improved reliability. Nickel monosilicide (NiSi) has proven to be a contact material of high technological interest^1^ and has been used in the 45 nm and the 32 nm transistors because it features one of the lowest resistivity of all silicides ($10.5\,{\rm{\mu }}\Omega \,{\rm{cm}}$), immunity to the fine-line effect, low formation temperature and low Si consumption^2^.

It is known that the transition route of the Ni-Si system is from Ni_2_Si to NiSi and subsequently to NiSi_2_ at the temperature of 800 °C^3^. However, the current extremely scaled transistor generations demand silicide film with thickness <10 nm^1^. In this sub-10 nm regime, the full understanding of the silicide phase transitions has not yet been achieved. For example, there is a critical thickness of the initial Ni films below which the transition route is completely altered. This phenomenon was first reported in the studies of Tung *et al*.^4^, in which the Ni films of $1\mbox{--}30\AA$ were found transformed to epitaxial layers on Si(111) and Si(100) substrates after annealing to 450 °C. Surface-sensitive methods, such as Auger electron spectroscopy and low energy electron diffraction, indicated the epitaxial phase to be NiSi_2_. Subsequent studies using transmission electron microscopy suggested that the epitaxial films is non-stoichiometric, in which the Ni/Si ratio is about $1/1.5\mbox{--}1.7$^5^. Another study suggested that the epitaxial layer might consist of small domains (a few nanometer) of both NiSi_2_ and hexagonal θ-nickel silicide with variable Si composition of 33–41%^6^.

As for the characterization of the materials, the commonly employed techniques are pole figure measurements (PFM)^6,7^, transmission electron microscopy (TEM)^8,9^ and Rutherford backscattering spectrometry (RBS)^10,11^. For example, in the PFM one acquires the diffraction response from the crystallites, hence providing the types of phases and the textural information. As the penetration depth of the X-ray used in the PFM is several microns, depth information of the silicide films is unavailable. TEM and RBS can provide in principle the depth-resolved composition and crystallography. However, as the thickness of silicide films is continuously decreasing, *in-situ* real-time characterization with high depth resolution of the films is becoming challenging for all of the above methods. This problem is one of the contributing factors for the spread of reported data of the ultrathin films. As of now, several basic questions remain open, such as: what is the composition of the epitaxial silicides and at which temperature does the epitaxial phase start to form?

Ion scattering, most commonly RBS, has been a particularly helpful tool for characterization of thin films, due to its high intrinsic accuracy^12^. Typically, a mono-energetic ion beam (usually helium or hydrogen ions) is scattered from the target. Through elastic scattering from target nuclei, the ions lose kinetic energy, depending on the atomic mass of the target nuclei. The ratio between scattered energy (E_1_) and initial energy (E_0_) is called kinematic factor, which can be calculated analytically for any pairs of projectile and target nucleus and thus permits to identify the elements present in a sample. Likewise, the probability of a scattering event between projectile and the target atoms, i.e. the scattering cross-section, can be calculated analytically enabling accurate quantification. Along their path in the solid, the ions gradually lose additional energy due to electronic interactions. The amount of lost energy per path length is called electronic stopping power (dE_e_/dx). With stopping powers obtained from available databases^13^, one can estimate the thickness of the thin films. Finally, in case of highly ordered materials, the parallel ion beams can channel in crystal axes or planes, revealing the characteristics of the crystals such as the structures, the lattice locations and defects in real space^14^. Ion scattering techniques are generally considered non-destructive, as the irradiation doses of the measurement are usually three orders of magnitude smaller than the amorphization thresholds^15^.

As compared to the conventional ion scattering operated at ${\rm{MeV}}$ primary energies, Medium-Energy Ion Scattering (MEIS) can provide the depth resolution of a few nanometers^16–18^. At medium energies, detection of particles using systems different from semiconductor detectors is possible. Hence, the energy discrimination is greatly enhanced. Examples are electrostatic, magnetic spectrometers and multi-channel-plate delay line detectors (MCP-DLD). In combination with a pulsed beam, the latter can provide a large area position-sensitive detector permitting to acquire the X-Y positions and the energies of every particle hit (hence, the term 3D). This is very beneficial because the blocking patterns, i.e. angular-dependent yield of the backscattered particles, in a selected range of energy can be acquired and permits to perform depth-resolved crystallography in real space^14,19,20^. Thanks to the large-area detector and the more efficient detection mechanism, 3D-MEIS can be considered even less invasive than RBS because the required doses are commonly two orders of magnitude smaller than in RBS.

## Experiments

In this report, we demonstrate an *in-situ* study simultaneously providing the depth-resolved composition and the real-space crystallographic information of ultrathin nickel silicide films ($< 10\,{\rm{nm}}$) using 3D-MEIS. Two sets of samples with two different initial thicknesses were used: 10 nm and 3 nm Ni films on Si (100) substrates. At first, the Si substrates were cleaned using 0.5% HF solution to remove the native oxide and then immediately loaded into a magnetron sputtering tool (von Ardenne CS730S). The substrates were deposited with the Ni films using the pulse direct current magnetron sputtering: $150\,{\rm{W}},\,6\times {10}^{-3}\,{\rm{mTorr}}$ Ar atmosphere and deposition rate $0.5\,{\rm{nm}}/{\rm{s}}$ After the deposition, accurate thickness measurements using RBS showed the Ni thickness to be 10.3 nm and 2.7 nm, respectively (assuming bulk density). The *in-situ* study was done with a 3D-MEIS system at the Uppsala University^21,22^. This system is equipped with a MCP-DLD detector (DLD120) from RoentDek. The detector is $\sim 120\,{\rm{mm}}$ in diameter and located at a variable circular position 290 mm away from the samples, forming a circular acceptance angle of 21.9°. The scattering chamber is equipped with a 6-axis goniometer with 3 translational and 3 rotational degrees of freedom, orthogonal on each other, respectively. The heating filament is located underneath the samples, whereas a k-type thermocouple is placed in contact with the sample surface. The temperature reading was calibrated using the melting points of two metals, indium (T_m_ = 156.6 °C) and lead (T_m_ = 327.5 °C).

When evaluating the backscattering spectra, we use SIMNRA for the simulation^23^. This program package usually considers single scattering events, in which the trajectories of the incoming and the outgoing ions are otherwise straight. This simplification is, most of the time, physically correct, and enables us to use analytical formulae for the calculation and hence, speeds up the simulation. Deviation from the single-scattering approximation might happen in the lower energy regime and for heavy projectiles and target elements. The travelling ions can experience small deflections with small scattering angles (multiple scattering) or more-than-one scattering events with large scattering angles (plural scattering). SIMNRA also provides options to tackle multiple and dual scattering and produces a better fit to the background of the measurements. These two options were chosen in evaluating our data. Nevertheless, plural scattering can be more significant at very low energy (tens of keV), and is accurately treated only with more demanding Monte Carlo simulation programs, such as the TRIM for backscattering (TRBS) code^24^. To assess the contribution of plural scattering to our measurements, we simulate backscattering spectra for our measurements using both programs. For the nickel silicide systems, our comparisons of the SIMNRA and the TRBS gives excellent agreement for spectra at $50\,{\rm{keV}}$ and $100\,{\rm{keV}}$ primary He^+^ ions, thus the faster simulations were used for fitting the present data.

## Results

**Figure 1:**
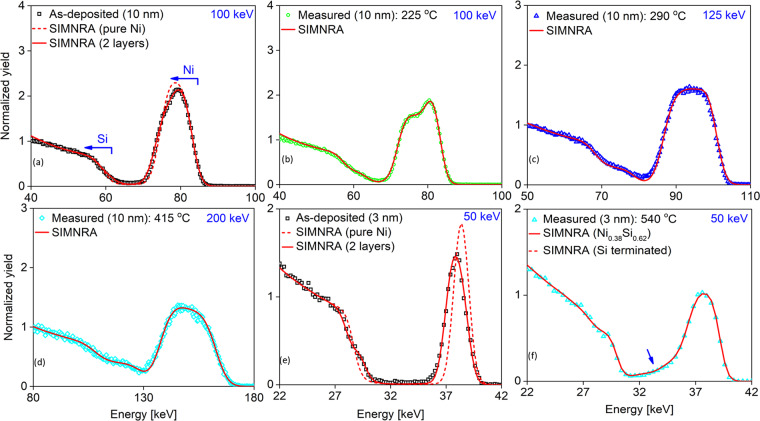
Energy spectra of He backscattered from 10 nm (**a**–**d**) and 3 nm Ni on Si (100) samples (**e**,**f**) annealed at increasing temperatures. Data points represent measurements and the solid lines are simulations using SIMNRA. The primary energy of the ion beam is indicated with the blue texts at the top right corner.

Figure 1 presents the spectra obtained for the 10 nm (a-d) and the 3 nm Ni films (e,f) annealed at increasing temperatures. The spectra were recorded for helium primary ions at $100\,{\rm{keV}}$ (a,b), $125\,{\rm{keV}}$ (c), $200\,{\rm{keV}}$ (d) and $50\,{\rm{keV}}$ (e,f). The energies were chosen to optimise the depth resolution while still be able to resolve the thickness of the whole films. For the composition measurement, the incidence and exit angles of the beam was set to avoid any crystal axes and planes of the crystal (complete random geometry), which is a necessary condition for the employed simulation codes. Experimental data are presented as open circles, whereas dash and solid lines are the corresponding simulation using SIMNRA. The electronic stopping power of the nickel, silicon and nickel silicide for the simulation is taken from our recent evaluation of the stopping power in the Ni-Si material system^25^. It is worth noting that an uncertainty of $4\mbox{--}5{\rm{ \% }}$ should be expected for the evaluated results. This uncertainty originates from a small inevitable portion of the beam channelling in the crystal^26^, the error of the employed stopping power and the counting statistics of the measurement.

The general features of all backscattering spectra can be qualitatively interpreted according to the introduction of the ion scattering technique in the previous section. From high to low energy, the first rise in the scattering yield is due to the scattering with the heavier element, nickel (the blue arrow in Fig. 1(a) indicates the kinematic limit). The finite width and height of the peak represent the depth distribution and the composition of nickel, respectively. The following rise of the scattering yield is due to the scattering with the lighter element, silicon accordingly. Figure 1(a) is the spectrum of the as-deposited $10\,{\rm{nm}}$ Ni film. As compared to the simulation of a homogenous pure film (red-dash line), the measured spectrum features a cut-off at the top and a significant skewness of the low-energy edge of the Ni peak. This observation indicates an intermixing layer between Ni and Si at the interface. Accordingly, a two-layer model (red-solid line), in which the top layer is pure Ni and the second layer consists of 68% Ni, provides a much better fit to the measurement. The formation of an intermixing layer between the deposited metals, such as gold^27^, platinum^28^ and Ni^29^, is a well-known phenomenon in both sputtering and electron beam evaporation process.

In Fig. 1(b), the necessity for a two-layer model to be used in the SIMNRA simulation becomes even more apparent. The sample, for which this spectrum was recorded, was annealed to 225 °C at a rate of 25 °C/min and then kept on hold for $1\,{\rm{\min }}$. In the simulation shown, the top layer is of pure Ni and $\sim 4.4\,{\rm{nm}}$ thick. Whereas, the Ni:Si ratio of the second layer is close to 2:1, in agreement with previous reports that the Ni_2_Si is the first phase of the Ni-Si transitions in the samples having bulk characteristics^2^. The thickness of the second layer results in $\sim 9.5\,{\rm{nm}}$, assuming bulk densities. At an annealing temperature of 290 °C, the Ni peak becomes more uniform (Fig. 1(c)). Employing SIMNRA to fit this spectrum shows that the silicide phase is dominantly Ni_2_Si with a thickness of $\sim 15\,{\rm{nm}}$. At 415 °C the silicide phase is determined to be a homogenous NiSi layer (Fig. 1(d)) with a thickness of $\sim 22\,{\rm{nm}}$. For recording the spectra of the 3 nm Ni samples (Fig. 1(e,f)), the primary beam energy was reduced to $50\,{\rm{keV}}$ to improve the depth resolution of the measurements. In the spectrum recorded for the as-deposited $3\,{\rm{nm}}$ film of Ni, a model assuming a layer of pure Ni (red-dash) cannot fully reproduce the measured data. As for the $10\,{\rm{nm}}$ sample, a two-layer model (red-solid), which includes an intermixing layer at the interface, provides a more accurate fit to the measurement. Finally, Fig. 1(f)shows the spectrum of the $3\,{\rm{nm}}$ sample annealed at 540 °C. The simulated spectrum using SIMNRA shows that a uniform silicide layer has formed with a composition of $\sim 38 \%$ Ni and $\sim 62 \%$ Si. There is also a subtle amount of Ni ($\sim 4 \%$) diffusing deeper into the Si substrate (blue arrow).

**Figure 2:**
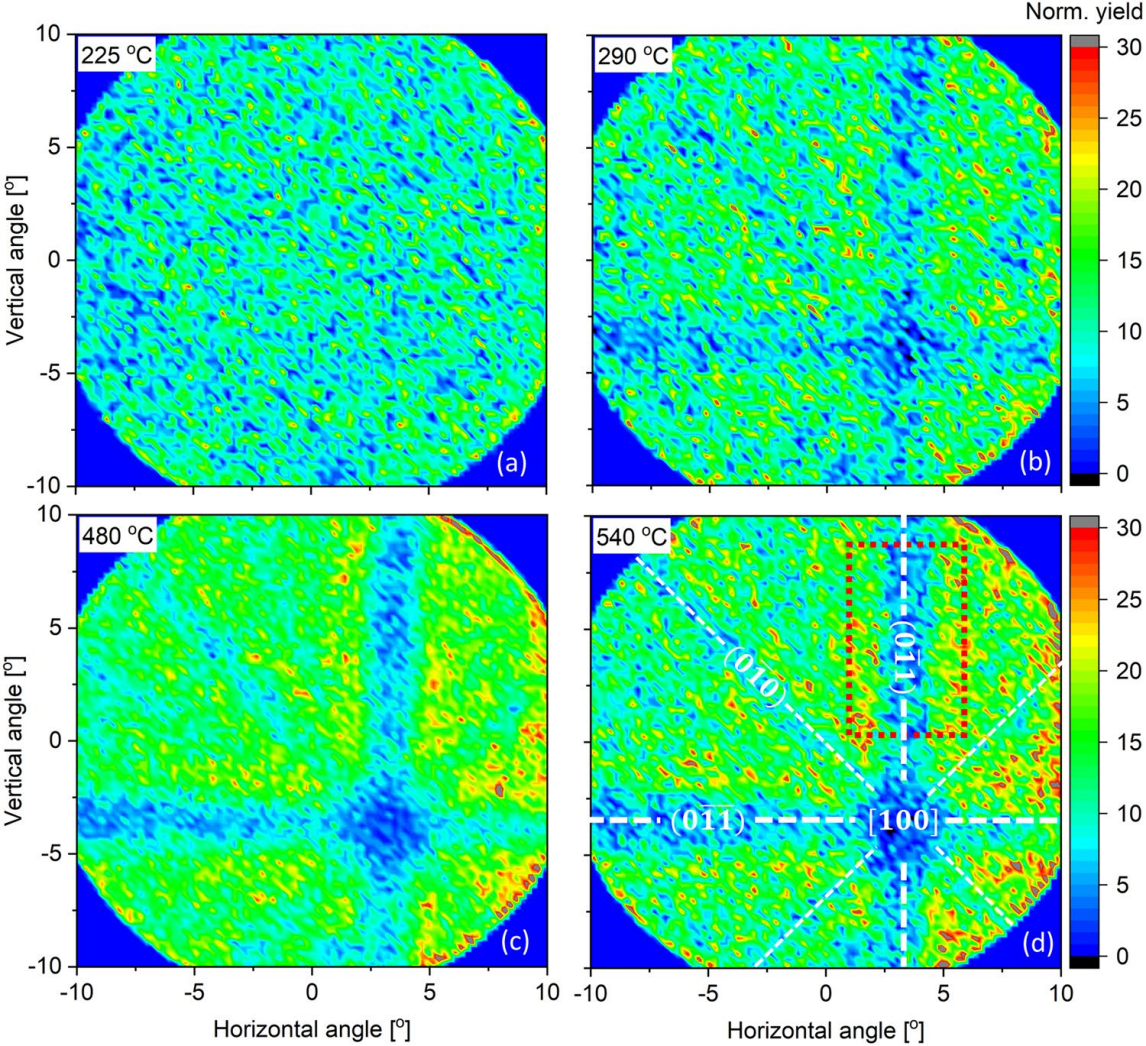
Blocking patterns of the ions scattered from the $3\,{\rm{nm}}$ Ni on Si $(100)$ samples annealed at 225 °C (**a**), 290 °C (**b**), 480 °C (**c**), and 540 °C (**d**). The patterns shown origin from ions with scattered energies of $70\mbox{--}85\,{\rm{keV}}$, equivalent to ions scattered from Ni atoms.

To obtain a more detailed picture of the transition of the $3\,{\rm{nm}}$ Ni films we acquired blocking patterns of the scattered ions on the detector employing the He ion beam at $100\,{\rm{keV}}$ primary energy. For that purpose, the sample normal was aligned close to the detector normal and the beam was incident at 40° off the sample normal. Figure 2 shows the blocking patterns of the scattered ions with the energy of $70\mbox{--}85\,{\rm{k}}{\rm{e}}{\rm{V}}$. This energy window corresponds to ions scattered from Ni atoms in the entire silicide layer, according to the energy spectrum for the same sample (Supp. Fig. 1). For the sample annealed at 220 °C for 1 min (Fig. 2a), the scattered ions are distributed uniformly on the detector, indicating a macroscopically disordered atomic arrangement in the Ni-Si layer. At a temperature of 290 °C, a blocking pattern starts to appear, suggesting a more orderly arrangement of the atoms in the silicide layer. This pattern becomes significantly more pronounced at elevated temperature of 480 °C and 540 °C. The evolution of the blocking patterns demonstrates that the sputtered Ni layer was initially disordered, supposedly polycrystalline or amorphous, up to 225 °C. The film then starts to crystallise at the temperature of 290 °C. Furthermore, in the Supplementary section we show the blocking pattern of the ions with the energy of $30\mbox{--}60\,{\rm{k}}{\rm{e}}{\rm{V}}$, i.e. the ions scattered from the Si atoms (Supp. Fig. 2). As a comparison, the patterns of the Si substrate and the silicide layer are similar. No noticeable difference in the position of the major axis [100] and its related planes between the two patterns. This similarity shows that the crystal axis and the planes of the silicide layer are very-well aligned with those of the silicon substrate. In other words, the silicide layer grows epitaxially on the Si substrate.

**Figure 3:**
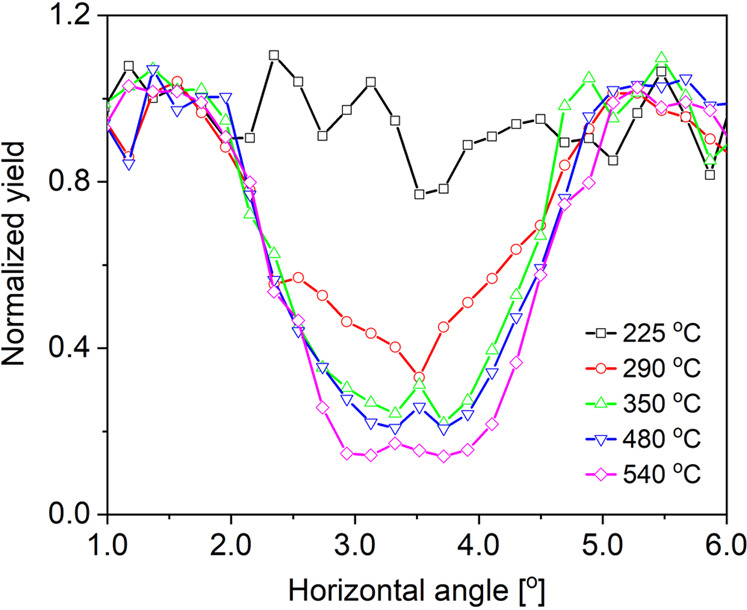
Scattered yields as a function of the horizontal angle as integrated within the box in the Fig. 2(d).

To allow for a quantitative assessment of the crystallinity, Fig. 3 shows the integrated yields within the red-dot box in Fig. 2(d). The dip in the scattering yield at 290 °C is found shallower as compared to the one at 540 °C. This observation can be either associated with the overall crystal quality of the film to be quite poor or the crystallization at low temperatures is starting only from the interface at 290 °C, while the overlaying layer is still disordered. The latter case is expected as it is found that low temperature reaction between Ni and Si can form a few epitaxial monolayers at the interface while the overlayer is still disordered^30^. At 350 °C, the crystallization is significantly more apparent and keeps improving at elevated temperature

The temperature-dependent results of the sample crystallography can be compared to earlier studies using different methods. Using AES, Tung *et al*. found that the epitaxial layer after the thermal annealing of $1\mbox{--}30\AA$ Ni films on Si to be NiSi_2_^4^. The formation temperature of this layer was reported to be 450 °C Other studies using *in-situ* XRD pole figure measurement suggested that the epitaxial layer formed at 500 °C^31^. These temperatures are considerably higher than in our study. However, as AES is a surface-sensitive technique, it can detect the NiSi_2_ phase only when the whole layer is transformed. Whereas, in the pole figure measurement the epitaxial layer is so thin that it is deemed difficult to detect the diffraction signal and hence the determination of the accurate temperature for the NiSi_2_ phase might be compromised. These results show the advantages of MEIS in the study of depth-resolved real-space crystallography of the ultra-thin films because ion beam channelling is very sensitive to the near-surface crystal arrangement.

## Conclusion

In summary, we have demonstrated the employment of the 3D-MEIS in studying *in-situ* the phase transition of ultrathin nickel silicides $({\rm{t}} < 10\,nm)$ during thermal annealing. The advantages of the approach are the depth-resolved composition and real-space crystallography in the nanometer regime in combination with a non-invasive probe. Using the position-sensitive detector, we can, for a single sample, *in-situ* and stepwise record the transition of the initially disordered Ni film to an epitaxial layer on the Si substrate. Prior to its full transition, the epitaxial layer is expected to form only at the interface at 290 °C. The silicide layer is fully epitaxial at the temperature of 540 °C and has the composition of 38% Ni and 62% Si.

## Supplementary information

Supplementary information.
