# Supplementary material for: In-situ characterization of ultrathin nickel silicides using 3D medium-energy ion scattering
Source: Sci Rep. 2020 Jun 24;10:10249. doi: 10.1038/s41598-020-66464-1 (PMC7314745; doi:10.1038/s41598-020-66464-1)
Supplement: Supplementary file 1 — Supplementary information. [file 41598_2020_66464_MOESM1_ESM.docx]

***In-situ* characterization of ultrathin nickel silicides using 3D medium-energy ion scattering**

Tuan Thien Tran ^a^, Lukas Jablonka ^b^, Christian Lavoie ^c^, Zhen Zhang ^b^, and Daniel Primetzhofer ^a^

*^a^ Department of Physics and Astronomy, Ångström Laboratory, Uppsala University, Box 516, SE-751 20 Uppsala, Sweden*

*^b^ Solid State Electronics, The Ångström Laboratory, Uppsala University, SE-75121 Uppsala, Sweden*

*^c^ IBM Thomas J. Watson Research Center, Yorktown Heights, New York 10598, USA*

Corresponding author: Tuan Thien Tran ([tuan.tran@physics.uu.se](mailto:tuan.tran@physics.uu.se))


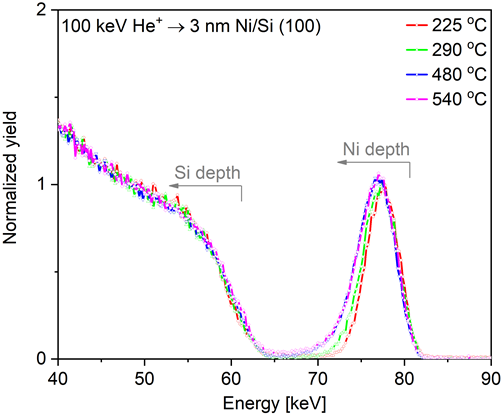


Supp. Fig. 1: Backscattering spectra of the 3 nm Ni-on-Si samples as annealed at increased temperature. Ions with the energy smaller than 65 keV are scattered from the Si atoms. Whereas, ions within the energy window of 70 – 85 keV are from the heavier Ni atoms. The ions scattered from the Ni atoms were chosen for the construction of the blocking patterns shown in Fig. 2.


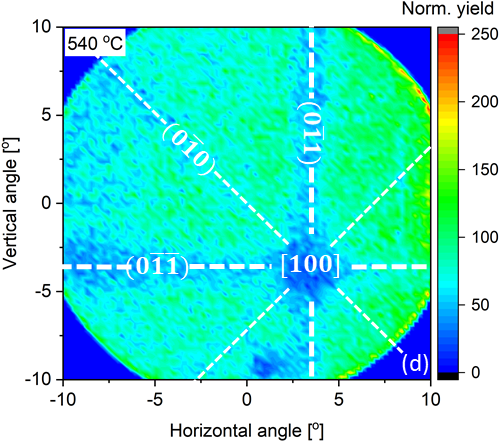


Supp. Fig. 2: Blocking pattern of the scattered ions with the energy of 30 – 60 keV, i.e. from the Si substrate. The patterns from the Si substrate and the silicide films of Fig. 2(d) are mostly similar, such as the position of the major axis [100] and the related planes. This similarity unequivocally shows that the crystal axis [100] and related planes are very-well aligned with those of the Si lattices, i.e. the silicide layer is crystalline and epitaxial on the Si substrate.
